# Novel Anti-Acanthamoebic Activities of Irosustat and STX140 and Their Nanoformulations

**DOI:** 10.3390/antibiotics12030561

**Published:** 2023-03-13

**Authors:** Ruqaiyyah Siddiqui, Mutasem Rawas-Qalaji, Mohammed I. El-Gamal, Sreedevi Sajeev, Jayalakshmi Jagal, Seyed-Omar Zaraei, Rawan M. Sbenati, Hanan S. Anbar, Wolfgang Dohle, Barry V. L. Potter, Naveed Ahmed Khan

**Affiliations:** 1 College of Arts and Sciences, American University of Sharjah, University City, Sharjah 26666, United Arab Emirates; 2 Department of Medical Biology, Faculty of Medicine, Istinye University, Istanbul 34010, Turkey; 3 Sharjah Institute for Medical Research, University of Sharjah, Sharjah 27272, United Arab Emirates; 4 Department of Pharmaceutics and Pharmaceutical Technology, College of Pharmacy, University of Sharjah, Sharjah 27272, United Arab Emirates; 5 Department of Medicinal Chemistry, College of Pharmacy, University of Sharjah, Sharjah 27272, United Arab Emirates; 6 Department of Medicinal Chemistry, Faculty of Pharmacy, Mansoura University, Mansoura 35516, Egypt; 7 Department of Clinical Sciences, College of Medicine, University of Sharjah, Sharjah 27272, United Arab Emirates; 8 Department of Clinical Pharmacy and Pharmacotherapeutics, Dubai Pharmacy College for Girls, Dubai 19099, United Arab Emirates; 9 Medicinal Chemistry and Drug Discovery, Department of Pharmacology, University of Oxford, Mansfield Road, Oxford OX1 3QT, UK

**Keywords:** *Acanthamoeba castellanii*, CNS infections, free-living amoebae, irosustat, PLGA nanoparticles, STX140

## Abstract

Pathogenic *Acanthamoeba* produce keratitis and fatal granulomatous amoebic encephalitis. Treatment remains problematic and often ineffective, suggesting the need for the discovery of novel compounds. For the first time, here we evaluated the effects of the anticancer drugs Irosustat and STX140 alone, as well as their nanoformulations, against *A. castellanii* via amoebicidal, excystment, cytopathogenicity, and cytotoxicity assays. Nanoformulations of the compounds were successfully synthesized with high encapsulation efficiency of 94% and 82% for Irosustat and STX140, respectively. Nanoparticles formed were spherical in shape and had a unimodal narrow particle size distribution, mean of 145 and 244 nm with a polydispersity index of 0.3, and surface charge of −14 and −15 mV, respectively. Irosustat and STX140 exhibited a biphasic release profile with almost 100% drug released after 48 h. Notably, Irosustat significantly inhibited *A. castellanii* viability and amoebae-mediated cytopathogenicity and inhibited the phenotypic transformation of amoebae cysts into the trophozoite form, however their nanoformulations depicted limited effects against amoebae but exhibited minimal cytotoxicity when tested against human cells using lactate dehydrogenase release assays. Accordingly, both compounds have potential for further studies, with the hope of discovering novel anti-*Acanthamoeba* compounds, and potentially developing targeted therapy against infections of the central nervous system.

## 1. Introduction

*Acanthamoeba castellanii* is a free-living protist pathogen belonging to the Genus *Acanthamoeba*. Based on 18S rDNA sequencing, Genus *Acanthamoeba* has been classified into 23 genotypes, i.e., T1–T23, but isolates belonging to the genotype T4 have most commonly been associated with the infections [1]. However, it is unclear whether this is due to the increased pathogenicity traits of the isolates belonging to the T4 genotype or their wide distribution in the environment that leads to more exposure to this genotype. Given a wide distribution of amoebae in the environment, including soil, water, and even air, globally, it is considered as one of the most ubiquitous protists, and humans and animals are often exposed to this organism [2,3,4]. However, despite their ubiquity, the number of infections due to pathogenic *Acanthamoeba* are relatively low, indicating the opportunistic nature of *Acanthamoeba*. It is now associated with producing sight-threatening *Acanthamoeba* keratitis that is often associated with the improper use of contact lenses, as well as fatal brain infection, often associated with weaker immune response and/or predisposition such as AIDS, steroid therapy due to organ transplantation, diabetes, etc. [2,3,4,5]. With global warming and increased outdoor water-related activities as well as contact lens use for vision correction and cosmetics, it is expected that the number of infections due to pathogenic *Acanthamoeba* will continue to rise [5,6]. *Acanthamoeba* exists in two forms; an infective trophozoite form during which *Acanthamoeba* divides vegetatively via binary fission, and a cyst form, which is a dormant and inactive form [2,3,4]. Notably, cysts do not cause damage to host cells and remain dormant to withstand harsh conditions such as lack of food, presence of antimicrobials, extreme temperatures/pH/osmolarity, etc. Cysts must re-emerge as viable trophozoites to produce damage to host cells resulting in infection. Currently available anti-*Acanthamoeba* drugs are often repurposed antimicrobials and/or antitumor compounds that exhibit inconsistent efficacy, due to the ability of *Acanthamoeba* to develop drug resistance [7,8,9]. The mixture of drugs includes chlorhexidine, diamidines, miltefosine, azole compounds, etc. [10]. However, miltefosine has been identified as an effective antiamoebic compound of therapeutic value [11,12]. It belongs to the class of alkylphosphocholine drugs, which are phosphocholine esters of long-chain aliphatic alcohols. It has been used effectively against *Leishmania* and the mode of action involves the impairment of acidocalcisome function and the activation of the sphingosine-dependent plasma membrane Ca^2+^ channel [13].

Furthermore, the ability of cysts to switch phenotypically and re-emerge as infective trophozoites leads to disease recurrence and presents a problem in the successful prognosis. Hence, there is a need for novel antiamoebic drugs that can effectively eradicate amoebae.

**Figure 1:**
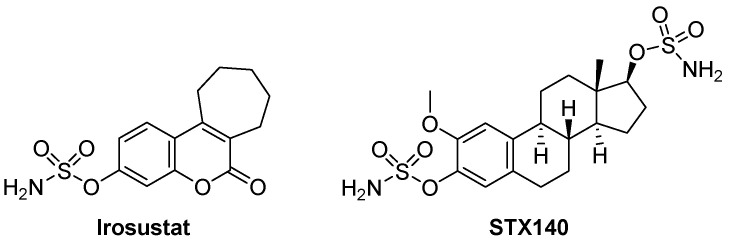
Structures of Irosustat and STX140.

Irosustat and STX140 (Figure 1) are sulfamate derivatives that have been reported as antiproliferative agents by inhibiting steroid sulfatase with demonstrable activity against cancer cells. Irosustat has been tested against prostate, endometrial, and breast cancers [14,15,16] and STX140 [17] is a candidate drug for hormone-independent cancers. Sulfate activation is prevalent in bacteria, protozoa, fungi, plants, and metazoan [18]. Furthermore, activation via sulfatases is known to play a role in the metabolism of *Entamoeba* and so it is logical to propose that it plays a role in *Acanthamoeba* differentiation and/or metabolic activity [19]. Herein, for the first time, we tested their effects against pathogenic *A. castellanii* belonging to the T4 genotype. Additionally, drug-loaded nanoparticles (NP) were prepared to reduce undesired cytotoxicity [20,21]. Poly D, L-lactic-co-glycolic acid (PLGA) PLGA is FDA-approved and used to encapsulate drugs into nanoparticles [22]. Drug encapsulation into NP may result in one or more of several advantages, including enhancing drug solubility, sustaining and slowing down drug release, enhancing its cellular uptake, and shielding normal cells from drug cytotoxicity [20,21,23].

## 2. Results and Discussions

### 2.1. The Synthesis of Irosustat NP and STX140 NP Resulted in the Formation of Small Unimodal NP Size with High Drug Encapsulation Efficiency

The synthesized Irosustat and STX140 PLGA NP had a PSD of less than 250 nm (Z-average), which is a very good size to allow for cellular uptake, and a Pdi of 0.1 indicating a uniform and narrow unimodal PSD (Table 1) that would reduce drug encapsulation, release, and cellular uptake variability [24,25]. The zeta potential of the loaded NP was found to be negative in water and of a similar value for both drugs, representing the surface charge of PLGA forming the NP and indicating an efficient drug encapsulation rather than its adsorption on the surface of the formed PLGA NP. 

The encapsulation efficiency (EE%) of Irosustat and STX140 NP were 94% and 82%, respectively (Table 1). Higher EE% could be attributed to the proper encapsulation methods used and the drug’s hydrophobic nature, which easily interacted with the hydrophobic moiety of PLGA.

### 2.2. The Synthesis of Irosustat NP and STX140 NP Resulted in Uniform Smooth and Spherical Particles

**Figure 2:**
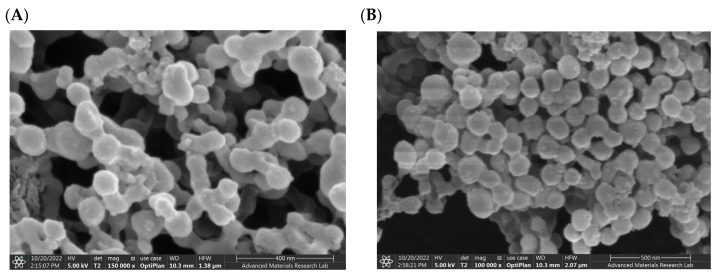
SEM images of synthesized PLGA NP: (**A**) SEM image of Irosustat (IRO) PLGA NP. (**B**) SEM image of STX140 (STX) PLGA NP.

The SEM photomicrographs of the drug-loaded NP revealed well-structured and well-formed spherical particles with a smooth surface (Figure 2). Spherical particles permit the greatest drug encapsulation capacity, which can be one of the contributing factors for high drug encapsulation reported earlier.

### 2.3. Both Irosustat and STX140 Resulted in a Rapid Drug Release from PLGA NP Followed by a Slower and More Controlled Release

The encapsulation of drugs into PLGA NP offers a biphasic drug release pattern characterized by an initial drug release followed by a more sustained released over time. The rate and the amount of released drug during the initial phase rely mainly on the properties of the selected polymer to synthesize the NP, such as its composition and molecular weight. The inherent lipophilicity of the encapsulated drug and the fabrication method of NP also play a detrimental role as well [20,21,26].

**Figure 3:**
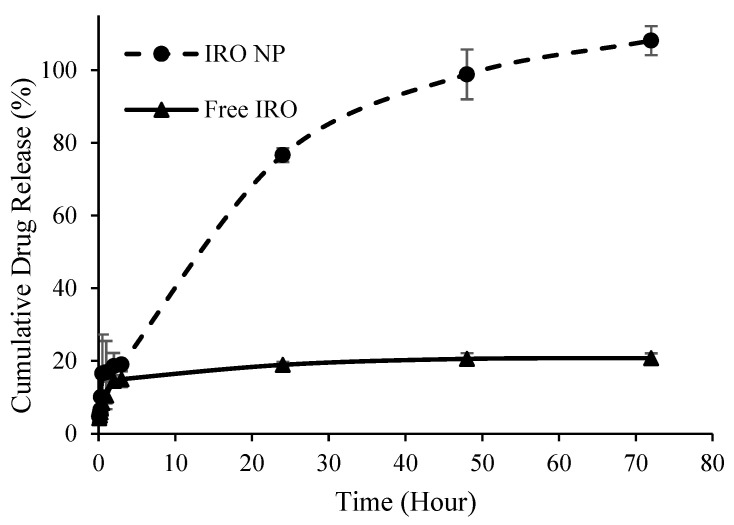
Cumulative Irosustat (IRO) release from PLGA NP over time versus free drug in DMSO.

**Figure 4:**
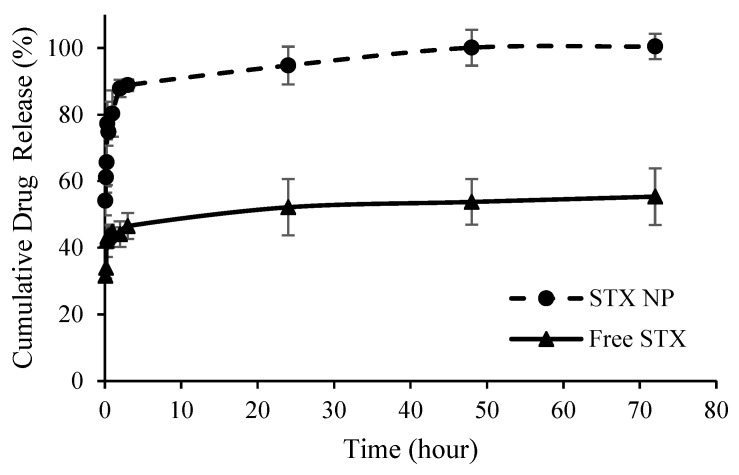
Cumulative STX140 (STX) release from PLGA NP over time versus free drug in DCM.

Similarly, the release of Irosustat and STX140 from PLGA NP followed similar release patterns but with different initial release rates (burst release). The maximum drug release was achieved after 72 h from both nanoformulations. The initial release was distinguished by a rapid release from the NP, with more than 75% drug released within 24 h, while the second release was characterized by a constant rate that continued for the next 72 h. For both Irosustat and STX140, almost 100% of the drug was released at 48 h compared to 20.6 ± 1.6% and 54 ± 6.7% from the solutions of free Irosustat in DMSO and free STX140 in DCM (Figure 3 and Figure 4).

The rapid drug release during the initial 24 h is likely due to the large surface area created by the successful synthesis of extremely small particles (Table 1). The increased surface area might facilitate more effective contact with the release medium and allowing for more rapid drug release and higher drug dissolution. Furthermore, a slower and more sustained drug release rate from the NP during the later release phase could be mainly due to the drop in the concentration gradient across the polymer membrane of the NP as a result of the release of most of the encapsulated drug and depletion of the drug in the reservoir system.

Furthermore, the large difference between the amount of drug released from the NP and the free drug that was almost constant over time was mainly due the significant enhancement in drug solubility at the nanoscale size due to the higher surface area, despite the use of a cosolvent to dissolve and detect the poorly soluble free drug in the aqueous solution.

### 2.4. Both Drugs Alone, as Well as Their Nanoformulations Displayed Significant Amoebicidal Activity against the Infective A. Castellanii Trophozoites Belonging to the T4 Genotype

**Figure 5:**
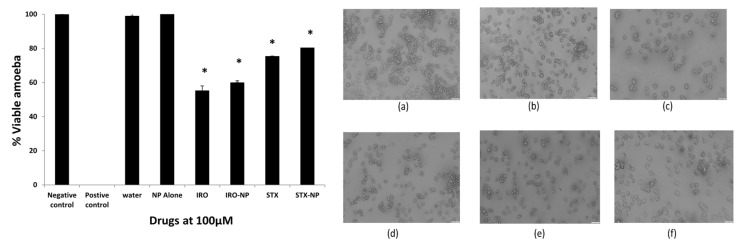
The amoebicidal activity of drugs was tested. All substances exhibited significant amoebicidal effects at the concentration of 100 µM post-24 h incubation against the *A. castellanii* trophozoites. Irosustat (IRO), STX140 (STX), and respective nanoparticles demonstrated significant amoebicidal activity against *Acanthamoeba castellanii*. The findings are shown as the mean ± sem (* represents *p* < 0.05). Microscopic analysis of *A. castellanii* after amoebicidal assay with Irosustat, STX140 and their respective nanoparticles: (**a**) amoeba alone with RPMI (negative control), (**b**) PLGA nanoparticles (placebo) along with amoeba—RPMI mix, (**c**) Irosustat along with amoeba—RPMI mix, (**d**) Irosustat nanoparticles along with amoeba—RPMI mix, (**e**) STX140 along with amoeba—RPMI mix, (**f**) STX140-NP along with amoeba—RPMI mix (200× magnification).

Amoebicidal experiments were carried out to determine whether the aforementioned drugs exhibit amoebicidal activity against the clinical isolate of infective *A. castellanii* trophozoites. At 100 µM dose, all compounds tested exhibited amoebicidal activity (Figure 5). The free Irosustat, Irosustat NP, free STX140, and STX140 NP reduced amoebic viability by 45%, 30%, 25%, and 19%, respectively, following a 24 h incubation period, while the NP alone (placebo NP) had no effect on amoebae viability. Overall, these findings showed, in comparison with the controls, that all compounds demonstrated significant amoebicidal action against *A. castellanii* at a concentration of 100 µM in 24 h (*p* < 0.05).

### 2.5. Irosustat and STX140 Exhibited Significant Effects against A. castellanii Excystment

**Figure 6:**
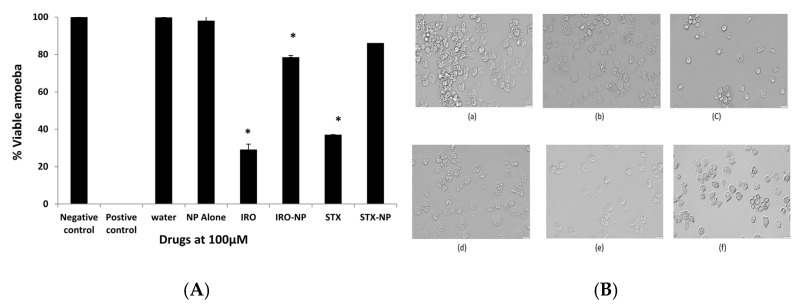
(**A**): Excystment experiments were conducted to examine the effects of the Irosustat (IRO), STX140 (STX), and their respective nanoparticles on *A. castellanii* excystation after noting the distinct impacts of Irosustat, STX140, and their respective nanoparticles. The findings are shown as the mean ± sem (* represents *p* < 0.05). (**B**) Microscopic analysis of *A. castellanii* after excystation assay with Irosustat, STX140, and their respective nanoparticles. (**a**) amoeba alone with RPMI (negative control), (**b**) PLGA nanoparticles (placebo) along with amoeba—RPMI mix, (**c**) Irosustat along with amoeba—RPMI mix, (**d**) Irosustat nanoparticles along with amoeba—RPMI mix, (**e**) STX140 along with amoeba—RPMI mix, (**f**) STX140-NP along with amoeba—RPMI mix (200× magnification).

To determine the effects of the aforementioned drugs against the transformation cysts into trophozoites, excystment assays were performed. As shown in Figure 6, the Irosustat and STX140 reduced amoebae excystation significantly (*p* < 0.05) as noted by the number of emerging trophozoites; however, the decrease shown by the STX140-NP was not statistically significant compared with the controls (Figure 6). In part, this could be due to slow drug release from their nanoformulations. The STX140 and Irosustat reduced *A. castellanii* excystment by around 71% and 63%, respectively, at 100 µM.

### 2.6. Irosustat NP and STX140 NP Exhibited Reduced Host Cell Cytotoxicity Compared with Their Free Drugs

**Figure 7:**
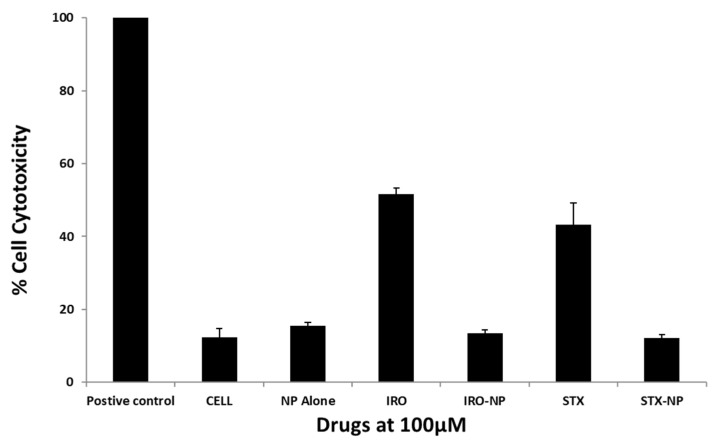
The toxicity of these chemicals towards human cells was assessed using cytotoxicity tests. All of the drugs used showed quite negligible cytotoxicity. The findings are shown as the mean ± sem.

Tests were conducted to determine the effects of the drugs on host cell damage. Notably, Irosustat and STX140 showed host cell cytotoxicity of approximately 51% and 43%, respectively. However, their nanoformulations exhibited host cell cytotoxicity of less than 20% (Figure 7). This is consistent with ISO 10993-5 which indicates limited cytotoxic activity if cell viability is between 60% and 80%; therefore, drug nanoformulations showed minimal toxic effects compared with the controls (Figure 7). In another experiment, we evaluated the cytotoxicity of Irosustat and STX140 against WI-38 normal lung cells. At 10 µM concentration, Irosustat and STX140 showed 38% and 32% growth inhibition, respectively. Furthermore, both compounds did not show 50% growth inhibition up to 20 µM.

### 2.7. Irosustat and STX140 Reduced A. castellanii Cytopathogenicity

**Figure 8:**
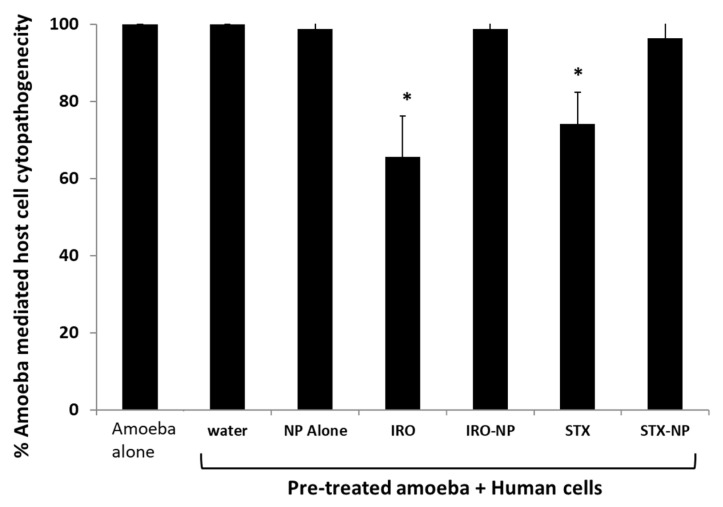
The effects of the Irosustat (IRO), STX140 (STX), and their respective nanoparticles on the cytopathogenicity mediated by *A. castellanii* were examined using cytopathogenicity assays. Notably, Irosustat and STX140 significantly decreased the cytopathogenicity of *A. castellanii.* The findings are shown as the mean ± sem (* represents *p* < 0.05).

Assays were performed to evaluate if both drugs can inhibit amoebae-mediated host cell damage. When compared with the negative control, Irosustat and STX140, but not their nanoformulations, reduced amoebae-driven human cell cytopathogenicity. Overall, human cell damage was reduced by over 35% and 25%, respectively within 24 h (Figure 8). In contrast, drugs encapsulated into PLGA NP did not affect amoebae-mediated host cell death. The limited effect of the drug nanoformulations could be due to slow drug release. Future studies will investigate the effects of drugs and drug nanoformulations over longer periods of time and identify their precise mode of action at the molecular level, followed by in vivo studies to determine the potential clinical efficacy of drugs.

*Acanthamoeba* are known to host a wide range of microorganisms including bacteria, viruses, fungi, and even other protists [27]. These interactions may contribute to varied virulence of the host amoebae as well as the endosymbiotic microbe. As *Acanthamoeba* can serve as a “genetic melting pot” where interspecies gene exchange may occur, it is plausible that different genotypes/strains of *Acanthamoeba* exhibit varied resistance to physical, chemical disinfectants, and pharmacological drugs. For example, previous studies have shown that endosymbionts may increase the pathogenicity of *Acanthamoeba* isolates and may have an effect on the virulent nature of amoebae and clinical characteristics of *Acanthamoeba* keratitis [28]. Thus, future work should also determine the effects of novel and/or modified antiamoebic compounds against different genotypes/strains of *Acanthamoeba* that may comprise different endosymbionts.

## 3. Materials and Methods

Poly D, L-lactic-co-glycolic acid (PLGA, 24,000–38,000 MW), polyvinyl alcohol (PVA), Kolliphor, Dimethyl sulfoxide (DMSO), Dichloromethane (DCM), and phosphate-buffered solution pH 7.4 (PBS) were obtained from Sigma.

### 3.1. Synthesis of Irosustat and STX140

Irosustat and STX140 were synthesized as previously described [29,30]. 

### 3.2. Preparation of Irosustat and STX140 Nanoparticles (NP)

Triplicates of Irosustat PLGA NP (*n* = 3) were synthesized by a previously reported nanoprecipitation process [20,21]. Briefly, 30 mg of PLGA was dissolved in 1 mL of DMSO, then 1 mg of Irosustat (equivalent to 108 μM) was added and vortexed for 1 min. The drug-polymer solution was added dropwise to 9 mL of Kolliphor aqueous solution (1% *w*/*v*) to nanoprecipitation. The formed Irosustat NPs were collected by centrifugation at 15,000× rpm for 20 min at 4 °C, and the separated NP pellet was washed with one mL of distilled water three times to eliminate residues of Kolliphor and non-encapsulated drug. The nanosuspension was centrifuged after each wash and water was discarded.

For the synthesis of STX140 NP, solvent evaporation approach was employed [31]. An amount of 1 mL of DCM mL was used to dissolve 190 mg of PLGA and 10 mg of STX140 by vortexing (*n* = 3). The resulting drug-polymer organic solution was added to 10 mL of 1% (*w*/*v*) PVA aqueous solution and sonicated for 5 min at 40% pulse with probe sonicator to form an emulsion. The created emulsion was quickly dropped into 125 mL of PVA (1%, *w*/*v*) under continuous stirring overnight to evaporate DCM and form STX140 NP. The generated NPs were then collected as a pellet by centrifugation for 30 min at 22,000× rpm and washed as before.

### 3.3. Determination of PSD, Pdi, and ZP 

The mean (Z-average) particle size distribution (PSD) and polydispersity index (Pdi) of the synthesized NP were established using dynamic light scattering and the photon correlation spectroscopy method at a scattering angle of 173°. The zeta potential (ZP), which measures the surface charge of the NP, was determined using the principles of laser Doppler velocimetry and phase analysis light scattering (M3-PALS technique). 

For the determination of PSD, pdi, and ZP at room temperature, a sample of 0.2 mL of freshly produced NP suspension after washing was diluted with 0.8 mL of distilled water and measured using Zetasizer Nano ZS (Malvern Instruments, Malvern, UK). Three measurements (*n* = 3) were used to determine the mean values and standard deviation.

### 3.4. Scanning Electron Microscopy

Scanning Electron Microscopy (SEM) (Thermo Scientific Apreo, FEI Company, Hillsboro, OR, USA) was used to study the shape, size, surface morphology, and topography of prepared NP. NP suspension samples were dried on SEM stubs. The samples were then gold-coated with a Quorum Technology Q150TS Sputter Coater (Ashford, Kent, UK) using Argon as the sputtering gas in a chamber with a pressure of 10^−2^ mbar, an applied voltage of 1 kV, and a plasma current of 18 mA for 120 s. At SEM acceleration voltage of 3 kV, the gold-coated samples were scanned, and photomicrographs were taken.

### 3.5. Determination of Drug Encapsulation Efficiency

The collected NP pellets were dissolved in DMSO or DCM to dissolve PLGA NP and release the whole entrapped drug and quantify their drug content by direct method. The drug absorbance of released and dissolved drug was measured at λmax 350 for Irosustat and λmax 300 for STX140 after scanning for their maximum absorbance using an ultraviolet (UV) spectrophotometer (Synergy H1, BioTek). Equivalent amounts of the PLGA polymer (without the drug) were dissolved in their corresponding solvents at the same previous concentrations to serve as a blank. Encapsulated drug from triplicates (*n* = 3) of dissolved NP was quantified using a standard calibration curve (R^2^ = 0.9) ranging from 0.08 to 5 mg/mL that was generated for each drug. The drug encapsulation efficiency (EE%) was calculated using the following equation:

$$\mathrm{Encapsulation}\;\mathrm{efficiency}\;\left(\%\right)=\frac{\mathrm{Encapsulated}\;\mathrm{Drug}}{\mathrm{Total}\;\mathrm{drug}\;\mathrm{added}\;}\times 100$$

### 3.6. In Vitro Release Studies

The collected NP pellets (*n* = 3) for each drug were dispersed in 1 mL deionized and transferred into dialysis tubing membranes (SpectraPor ^®^, 14 kDa molecular weight cut-off, Sigma-Aldrich, St. Louis, MO, USA). The dialysis membranes were sealed and immersed in 10 mL PBS of pH 7.4 and maintained at 37 °C as the release medium. Samples (100 μL) were withdrawn at various time intervals over 72 h. Withdrawn volumes were replenished with fresh release medium. Additionally, equivalent amounts of free Irosustat in 1 mL DMSO and free STX140 in 1 mL DCM were filled in dialysis bags and sampled as before. Drug content in the withdrawn samples was quantified using a UV spectrophotometer as described before, and the cumulative drug release percentage (mean ± SD) was calculated using the following equation:

$$\mathrm{Cumulative}\;\mathrm{drug}\;\mathrm{released}\;\left(\%\right)=\frac{\mathrm{Cumulative}\;\mathrm{amount}\;\mathrm{of}\;\mathrm{released}\;\mathrm{drug}}{\mathrm{The}\;\mathrm{total}\;\mathrm{amount}\;\mathrm{of}\;\mathrm{drug}\;\mathrm{in}\;\mathrm{nanoparticles}}\times 100$$

### 3.7. Culturing Acanthamoeba castellanii of the T4 Genotype

*Acanthamoeba castellanii* were purchased from the American Type Culture Collection (ATCC 50492). Amoebae were cultured in PYG (0.75% protease peptone, 0.75% yeast, and 1.5% glucose) at 30 °C in T-75 flasks as described previously [9,11,32,33,34]. In short, upon confluency, flasks were placed on ice for 10 min, with gentle tapping. Non-adherent amoebae were collected in 15 mL tubes and centrifuged at 2700× *g* for 5 min. Next, amoebae were enumerated using a hemocytometer and used for various experiments. 

### 3.8. Amoebicidal Assays against the A. castellanii Trophozoites of the T4 Genotype

Antiamoebic properties of drugs and drug-loaded NPs were determined against *A. castellanii* trophozoites as described previously [33,35]. In short, *A. castellanii* (5 × 10^5^ amoebae) were incubated with drugs, placebo NPs, and drug NPs in a 24-well plate with a final volume of 500 µL for 24 h at 30 °C. For negative control, amoebae were kept in RPMI alone whereas 0.25% sodium dodecyl sulfate (SDS) was used to obtain 100% kill. After this, Trypan blue (0.1%) was added to each well and viable amoebae were enumerated using a hemocytometer to determine viable amoebae. The basis of Trypan blue exclusion assay is that the dye penetrates the cell membrane of the dead parasites, and they appear stained (dark blue), while live parasites resist the dye and amoebae appear unstained.

### 3.9. Excystment Assays

To determine whether drugs and drug nanoformulations inhibit the ability of amoebae to switch from inactive cyst form to active trophozoite form, excystment assays were performed as described previously. Briefly, cysts were produced by placing amoebae trophozoites (approximately 1 × 10^7^) onto nutrient-free agar plates. Plates were incubated for up to 2 weeks at 30 °C and observed daily for the formation of cysts as described previously [32,33]. Next, 5 mL of phosphate-buffered saline (PBS) was added to each plate, and cysts were collected by gently scraping off cysts using a cell scraper. Next, cysts were collected in a 15 mL tubes and centrifuged at 3500× *g* for 10 min and then enumerated using a hemocytometer. Following this, drugs and drug nanoformulations were incubated with *A. castellanii* cysts in growth medium (PYG medium) to determine whether the drugs/drug nanoformulations inhibit the ability of amoebae to transform from the dormant cyst form to an active trophozoite form. Cysts were incubated in the growth medium for up to 72 h. During this time, cysts incubated in solvent alone emerged as viable trophozoites while the effects of drugs/drug nanoformulations were determined using hemocytometer counting. 

### 3.10. Cytotoxicity Assays

To determine the toxic effects of drugs and drug nanoformulations on human cells, cytotoxicity assays were performed as described previously [14]. Briefly, cervical cancer cells derived from Henrietta Lacks, known as HeLa cells, were procured from American Type Culture Collection (ATCC CCL-2) and cultivated in T-75 tissue culture flasks containing 10 mL of RPMI-1640, 1% Penicillin-Streptomycin (Pen-Strep), 1% minimum essential medium amino acids, and 1% L-glutamine in an incubator with 5% CO_2_ at 37 °C with 95% humidity. Once confluent, cells were grown in 96-well plates and used for various experiments as described previously [35]. For assays, HeLa cells were incubated with drugs and drug nanoformulations in 96-well plates overnight at 37 °C in a 5% CO_2_ incubator. Next, supernatants were collected, and lactate dehydrogenase release (LDH) was determined as described previously [35]. HeLa cells incubated with RPMI-1640 alone were used as negative controls. For positive control, 1% Triton X-100 was added to each well and cells were incubated for 45 min at 37 °C. Assays were performed in a 96-well plate by incubating an equivalent volume of LDH substrate and a similar volume of experimental cell supernatant. The plate was incubated in the dark at room temperature and then subjected to a multi-plate reader. At 490 nm, the absorbance was determined, and the percent cytotoxic effect was calculated using the following formula: % Cell cytotoxicity = ((Sample value − negative control)/(Positive control − negative control)) × 100.

WI-38 cell line was purchased from the European Collection of Cell Cultures (ECACC, Wiltshire, UK) and maintained in Roswell Park Memorial Institute medium (RPMI, Sigma-Aldrich, St. Louis, MO, USA). The medium was supplemented with 10% fetal bovine serum (FBS, Sigma-Aldrich) and 1% penicillin/streptomycin (Sigma-Aldrich). After treatment with the tested compounds or placebo, the cells were incubated at 37 °C in a humidified atmosphere of 5% CO_2_. 

The cells were seeded in 96-well tissue culture plates with a density of 4 × 10^4^/well and incubated overnight. The cells were then treated with the tested compounds for 48 h. DMSO (vehicle) was used as a negative control. The media were decanted following treatment, and the cells were incubated for 2 h at 37 °C with 200 μL media containing 0.5 mg/mL of MTT tetrazolium dye (Sigma-Aldrich). At the end, the media were removed and 200 μL of DMSO was added to solubilize the formed violet crystals. Absorbance was measured at 570 nm using a microplate reader (Thermo Scientific, Waltham, MA, USA).

### 3.11. In Vitro Cytopathogenicity Assays

To determine whether drugs and drug nanoformulations inhibit parasite-mediated host cell damage, in vitro cytopathogenicity assays were performed as described previously [34]. Briefly, *A. castellanii* (5 × 10^5^ amoebae) was challenged with drugs and drug nanoformulations for 2 h at 30 °C. Post-treatment, amoebae were centrifuged at 1500× *g* for 2 min, and pellet was re-suspended in 200 µL of RPMI-1640. Next, amoebae were inoculated on HeLa cells grown in 96-well plates for 24 h and then cell damage was determined by LDH assays as described above [33,34].

## 4. Conclusions

In conclusion, we successfully synthesized PLGA NP of both sulfamate-based Irosustat and STX140. Both drugs revealed significant amoebicidal, excystment activities and reduced the cytopathogenic of effects of *A. castellanii*. Of note, the drug nanoformulations depicted minimal cytotoxicity in comparison to the drugs alone. Therefore, Irosustat and STX140 have the potential to be evaluated further as potential candidates against *A. castellanii* infections. The mode of action of these drugs is via steroid sulfatase inhibition, but they also act more widely as multi-targeting agents [16,17], including activity on tubulin. Earlier studies also showed that Irosustat inhibited activity of CYP1A2 in liver microsomes, however Irosustat did not inhibit CYP2A6, CYP2B6, CYP2C8, CYP2C9, CYP2D6, CYP2E1, CYP3A4/5, or UDP-glucuronosyltransferase 1A1, 1A4, or 2B7 activities [36,37,38]. Similarly, several studies have shown the potential of STX140 as an anticancer agent as it targets steroid sulfatase and exhibits antiproliferative activities against a range of cancer cell lines and also inhibits angiogenesis [30,38,39,40,41,42,43]. Sulfatase activation is known to affect the metabolism of *Entamoeba* and may thus affect the differentiation and/or metabolic activity of *Acanthamoeba* and this should be investigated further [19]. Additionally, STX140 (compared with Irosustat) is more potent against a variety of cancer cell lines. Notably, as the free compounds and their nanoformulations inhibited excystment activity, they are of particular interest given the inhibition of the cysts is a useful avenue to target in order to prevent infection reoccurrence. Nonetheless, further research is needed to determine the precise mechanism of action in *A. castellanii.* Overall, these sulfamate derivatives should be evaluated in vivo to confirm their activity against *Acanthamoeba* and confirm their minimal toxicity. Moreover, the NP could in principle be modified (i) to enhance drug efficacy and/or (ii) for the targeted drug delivery in the central nervous system. Further studies are recommended to investigate the molecular mechanism of action of Irosustat and STX140 as antiamoebic candidates against *Acanthamoeba castellanii*.

## Figures and Tables

**Table 1:** Characteristics of synthesized IRO and STX nanoparticles (NP).

Formulation	Size (nm)	Pdi	Charge (mV)	EE (%)
IRO NP	145.2 ± 3.5	0.106 ± 0.02	−13.6 ± 0.709	94.2 ± 0.04
STX NP	243.6 ± 2.3	0.088 ± 0.01	−15.3 ± 0.808	82.34 ± 2.3

## Data Availability

The datasets generated or analyzed during the current study are available from the corresponding author upon reasonable request.
